# Irradiation induced inversions suppress recombination between the M locus and morphological markers in *Aedes aegypti*

**DOI:** 10.1186/s12863-020-00949-w

**Published:** 2020-12-18

**Authors:** Antonios A. Augustinos, Muhammad Misbah-ul-Haq, Danilo O. Carvalho, Lucia Duran de la Fuente, Panagiota Koskinioti, Kostas Bourtzis

**Affiliations:** 1 Insect Pest Control Laboratory, Joint FAO/IAEA Programme of Nuclear Techniques in Food and Agriculture, A-1400 Vienna, Austria; 2 Present address: Department of Plant Protection, Hellenic Agricultural Organization-Demeter, Institute of Industrial and Forage Crops, 26442 Patras, Greece; 3 Present address: Nuclear Institute for Food and Agriculture (NIFA), Peshawar, Pakistan; 4 Department of Biochemistry and Biotechnology, University of Thessaly, Biopolis, 41500 Larissa, Greece

**Keywords:** Chromosomal rearrangements, Population suppression, Sterile insect technique, Genetic sexing strains, Vector control

## Abstract

**Background:**

*Aedes aegypti* is the primary vector of arthropod-borne viruses and one of the most widespread and invasive mosquito species. Due to the lack of efficient specific drugs or vaccination strategies, vector population control methods, such as the sterile insect technique, are receiving renewed interest. However, availability of a reliable genetic sexing strategy is crucial, since there is almost zero tolerance for accidentally released females. Development of genetic sexing strains through classical genetics is hindered by genetic recombination that is not suppressed in males as is the case in many Diptera. Isolation of naturally-occurring or irradiation-induced inversions can enhance the genetic stability of genetic sexing strains developed through genetically linking desirable phenotypes with the male determining region.

**Results:**

For the induction and isolation of inversions through irradiation, 200 male pupae of the ‘BRA’ wild type strain were irradiated at 30 Gy and 100 isomale lines were set up by crossing with homozygous ‘red-eye’ (*re*) mutant females. Recombination between *re* and the M locus and the white (*w*) gene (causing a recessive white eye phenotype when mutated) and the M locus was tested in 45 and 32 lines, respectively. One inversion (Inv35) reduced recombination between both *re* and the M locus, and *w*and the M locus, consistent with the presence of a rather extended inversion between the two morphological mutations, that includes the M locus. Another inversion (Inv5) reduced recombination only between *w* and the M locus. In search of naturally-occurring, recombination-suppressing inversions, homozygous females from the red eye and the white eye strains were crossed with seventeen and fourteen wild type strains collected worldwide, representing either recently colonized or long-established laboratory populations. Despite evidence of varying frequencies of recombination, no combination led to the elimination or substantial reduction of recombination.

**Conclusion:**

Inducing inversions through irradiation is a feasible strategy to isolate recombination suppressors either on the M or the m chromosome for *Aedes aegypti*. Such inversions can be incorporated in genetic sexing strains developed through classical genetics to enhance their genetic stability and support SIT or other approaches that aim to population suppression through male-delivered sterility.

**Supplementary Information:**

The online version contains supplementary material available at 10.1186/s12863-020-00949-w.

## Background

*Aedes aegypti* is the primary vector of arthropod-borne viruses (arboviruses) such as dengue [1, 2], chikugunya [3], Zika [4, 5], and the yellow fever [6, 7]. It is one of the most widespread and invasive mosquito species globally, originated from Western Africa and spread worldwide in the past 70 years through human trading and travelling activities [8, 9].

Due to the lack of efficient specific drugs or vaccination strategies (except yellow fever) to impede disease transmission [10, 11], vector population control methods are receiving renewed interest. Main strategies so far mostly rely on the extensive use of insecticides and the community engagement for habitat management. However, both tools have been proven inefficient due to emerging insecticide resistance in *Ae. aegypti* populations, negative environmental and ecological impact of pesticide use, and difficulty in identifying and destroying mosquito breeding sites, particularly the cryptic ones [12–17]. Therefore, more effective, sustainable, and environmentally friendly control approaches are needed, including genetically based population suppression methods, such as the sterile insect technique (SIT) and other related methods, all of which rely mainly on the induction of sterility in natural populations.

Sterile males can be produced by irradiation-based (SIT), symbiont-based (incompatible insect technique, IIT), combined SIT/IIT or transgenic approaches [18–24]. An important requirement for any of them is the availability of an efficient and robust sex separation system that will result to the accurate separation of males from females [21]. Sex separation is currently possible in *Ae. aegypti* using mechanical tools based on pupal sexual dimorphism but their efficacy depends heavily on the rearing conditions [25–27]. Furthermore, these methods are currently appropriate only for small scale manipulations, hence it is essential to develop advanced sexing strategies based on genetic and molecular approaches for large scale mosquito male releases, including genetic sexing strains (GSS) [21, 26]. The development of a GSS through classical genetics requires two basic components: a) a visible or conditional recessive mutation that can be used as a selectable marker (e.g. eye or pupal color, insecticide resistance, etc.) and b) the linkage of the phenotypic marker to the sex-determining genetic locus [28]. There is a renewed interest in revival and application of SIT for mosquitos [24, 29–31], as evidenced also by recent advances in refinement of irradiation doses and drone-mediated releases in the field as well as the recently published guidance framework and phased conditional approach for testing the SIT against *Aedes* mosquitoes [32–34]. Such strategies will be greatly facilitated by the availability of GSS developed through classical genetics, since there is documented efficiency through time and wide public acceptance [35]. In addition, recent studies suggest that the impact of the irradiation on the biological quality of the males does not severely compromises their performance [31, 36, 37].

In *Aedes* mosquitoes, male development depends on a dominant male-determining locus (M-locus) that resides on a homomorphic sex-determining chromosome [38–40]. The M-locus has been mapped to chromosome 1, band 1q21 and the *Ae. aegypti* males are heterogametic (Mm) while the females are homogametic (mm) [41]. Recent studies suggest that the *Nix* gene has the properties of being the M-locus since it is both ‘required and sufficient to initiate male development’ [40, 42]. *Nix* resides in a genomic region that is protected from recombination and other functional genes have been identified tightly linked with *Nix,* with myo-sex being almost exclusively found in males and only sporadically in females, due to recombination [43]. An ideal selectable marker for *Ae. aegypti* would be located on chromosome 1, closely linked to M-locus. In order to construct a GSS, the wild-type allele of the selectable marker should be physically linked to the male-determining factor which in the Diptera model of GSS, the Mediterranean fruit fly, *Ceratitis capitata* (medfly), has been accomplished by Y-autosome translocation(s). In the resulting strain, the males are heterozygous with a normal “wild-type” phenotype. On the other hand, the females are homozygous for the recessive alleles of the selectable marker thus exhibiting the mutant phenotype and can be separated from males [28].

**Fig. 1:**
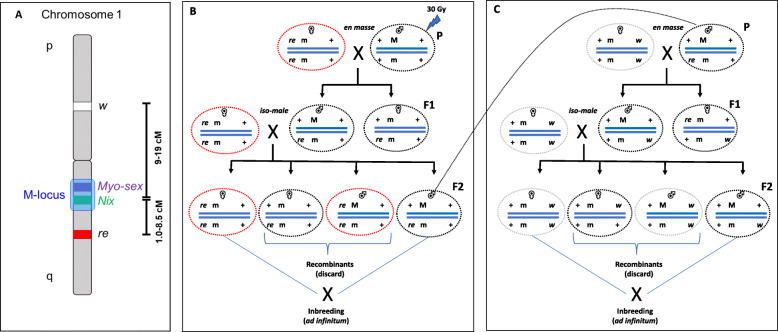
A schematic representation of the genetic distances in the *re-*M-*w* genomic region and the crossing scheme for the isolation of irradiation induced recombination suppressors in the same region. **a**: A schematic representation of the rough genetic distances between *re, w,* and M locus, as derived from the combination of previously reported findings. **b**: Induction of inversions through irradiation and isolation of recombination suppressors between *re* and the M locus; **c**: Isolation of recombination suppressors between *w* and the M locus

There is a wide range of potential markers known from previous studies that could be used for GSS development in *Ae. aegypti*. These are related to eye color [44–47], insecticide resistance [48, 49], and body color [50, 51]. Some of the eye color genes, such as the *red eye* (*re*) and the *white* (*w*), have already been mapped to the sex determining chromosome, which makes them suitable selectable markers since there is no need of inducing chromosome 1-autosome translocations [44, 45] (Fig. 1a). However, the presence of genetic recombination in males of *Aedes* species (unlike many other Diptera) would reduce the genetic stability of such a GSS.

Chromosomal inversions are quite common in *Aedes* species and are considered as an important factor contributing to speciation [52, 53]. Inversions can also be detected within species or populations undergoing speciation (incipient speciation) and small inversions can be widely spread in the genome. Intraspecies inversions can have multiple effects in respect to recombination frequencies and fitness [54–56]. In the case of homozygous inversions, where genomic regions of both homologous chromosomes are inverted compared to the ‘standard’ orientation, changes in recombination frequencies are expected, consistent with the effect of the inversion on the chromosomal localization of the markers studied. At the same time, no major negative effects on fitness are expected, unless inversion interferes with gene expression of important genes. On the other hand, heterozygous inversions (where a genomic region of one of the two homologues has been inverted) are considered as recombination suppressors, since the recombination is eliminated within the inversion, a phenomenon that can be extended also outside the inversion [54, 55]. Recombination suppression in this case mainly happens because recombination within the inversion leads to the production of imbalanced gametes, which are eliminated during development. Therefore, longer heterozygous inversions may have a cost on the fitness of a strain (in respect to productivity).

The construction of GSSs in the medfly has been facilitated by the fact that male recombination is highly suppressed [28]. The development of a filtering system for the mass rearing facilities further supported the adaptation of GSSs in large scale rearing [28, 57]. The incorporation of additional tools, such as the D53 inversion (Inv D53) further enhanced the genetic stability of the GSS [28, 58, 59]. However, in *Aedes* species, recombination frequency in males is not suppressed. Therefore, GSS developed through classical genetics may be unstable and additional elements that suppress recombination are needed. Recombination suppressing inversions have been developed in the past for both M and m chromosomes [60], leading to reduced recombination among the M locus and morphological markers of chromosome I.

The aim of the present study was to induce through irradiation (or identify naturally-occurring) inversions on the M chromosome of *Ae. aegypti* that would suppress recombination between *re* and M locus and/or *w* and M locus. These two morphological markers are being considered for the development of GSSs, therefore reducing recombination with M locus is critical for the enhancement of the genetic stability of resulting strains.

## Results

### Establishment of iso-male lines following irradiation

Approximately 300 male pupae of the ‘BRA’ strain were irradiated in batches of ~ 100 pupae each. Following the crossing scheme described in Methods section, one hundred iso-male lines were set up (Fig. 1b). Eggs from three consecutive gonotrophic cycles were collected and iso-male lines produced approximately 53 eggs per line (Additional file 1 Table S1). The first gonotrophic cycle produced most of the eggs (34.33 eggs per line), followed by the second (14.04 eggs per line) and the third (4.19 eggs per line). Only 57 of the one hundred lines produced eggs in at least one of the gonotrophic cycles. After excluding lines that produced zero eggs, the average number of eggs was 92.21 per line.

### Isolation of recombination suppressors (re - M)

Larvae hatched efficiently from 45 of the 57 lines that produced eggs (Table 1), allowing estimation of recombination frequency in F2 and, if needed, F3. As a rule of thumb and considering that recombination between the *re* and the M locus has been reported to vary between 1 and 7%, all lines that showed less than 2% percentage of recombinants in F2 were upscaled and evaluated again in F3. Therefore, thirteen lines were upscaled, as shown in Table 1. Ten of these lines did not exhibit reduced recombination after upscaling. Two lines (lines 17 and 19), showed evidence of reduced recombination both in F2 and F3 but could not be further sustained. However, one of the lines (line 35) showed significant suppression of recombination, since there were 0/221 recombinants in F2 and only 1/1257 in F3 (χ^2^ = 25.09; df = 1; p < < 0.001).

**Table 1:** Recombination frequencies between *re* and the M locus in 45 iso-male lines

Line	F	Genotypes					Recombination frequency
		Parental		Recombinant		Total	
		wt males	re females	re males	wt females		
5	F2	16	17	1	2	36	0.083
6	F2	15	26	0	0	41	0
	F3	92	55	6	4	157	0.064
9	F2	13	34	1	2	50	0.060
10	F2	9	1	0	0	10	0
	F3	6	3	0	1	10	0.1
16	F2	35	22	1	0	58	0.017
	F3	13	17	1	4	35	0.142
17	F2	21	21	0	0	42	0
	F3	4	7	0	0	11	0
19	F2	16	9	0	0	25	0
	F3	4	3	0	0	7	0
24	F2	2	4	0	0	6	0
31	F2	5	13	0	0	18	0
	F3	23	35	3	0	61	0.049
33	F2	12	42	1	2	57	0.052
34	F2	155	132	4	1	292	0.017
**35**	**F2**	**106**	**115**	**0**	**0**	**221**	**0**
	**F3**	**695**	**561**	**1**	**0**	**1257**	**0.0007**
37	F2	54	45	1	0	100	0.01
40	F2	21	22	0	0	43	0
41	F2	54	61	2	2	119	0.033
42	F2	30	19	0	0	49	0
43	F2	56	46	0	0	102	0
	F3	89	103	7	8	207	0.073
45	F2	47	33	1	0	81	0.012
48	F2	109	62	0	0	171	0
	F3	643	380	46	57	1126	0.091
49	F2	50	48	0	1	99	0.01
50	F2	31	27	0	0	58	0
	F3	216	156	6	2	380	0.021
51	F2	61	43	1	0	105	0.01
52	F2	74	89	1	2	166	0.018
54	F2	114	118	4	2	238	0.025
55	F2	46	34	13	0	93	0.139
57	F2	45	62	1	0	108	0.009
58	F2	35	36	0	0	71	0
	F3	369	184	24	22	599	0.077
59	F2	62	60	0	0	122	0
	F3	188	95	58	8	349	0.189
60	F2	33	23	1	11	68	0.176
61	F2	29	29	0	0	58	0
	F3	260	94	12	2	368	0.038
63	F2	6	7	2	0	15	0.133
64	F2	64	68	1	0	133	0.008
65	F2	4	5	0	0	9	0
67	F2	21	14	0	1	36	0.027
68	F2	33	23	1	0	57	0.017
71	F2	7	3	1	0	11	0.091
72	F2	22	12	0	0	34	0
75	F2	4	3	0	0	7	0
78	F2	26	14	0	0	40	0
82	F2	9	10	0	0	19	0
83	F2	8	4	2	1	15	0.2
91	F2	22	18	0	0	40	0
93	F2	32	26	0	2	60	0.033
94	F2	13	10	1	0	24	0.041
98	F2	14	17	0	0	31	0

In bold: line Inv 35 showing promising recombination-suppressing properties between *re* and *M* loci

### Isolation of recombination suppressors (w - M)

A total of 32 iso-male lines were successfully crossed and backcrossed with homozygous *w* mutant virgin females thus allowing the estimation of recombination between *w* and the *M* locus (Fig. 1b). Since recombination between these two loci has been reported to usually vary between 8 and 16%, lines that showed less than 6% of recombinants in F2 were evaluated again in F3. Only three lines (5, 35, and 67) gave less than 6% recombination in F2 (Table 2). One of the lines (67) showed increased recombination frequency in F3 and was not further followed up. Two of the lines showed consistent recombination suppressing properties. Line 5 showed recombination frequencies of approximately 1.4% in generations F2 and F3, with a rather large sample size tested (more than 1000 individuals per generation) (χ^2^ = 250.25; df = 1; p < < 0.001). Even more promising, line 35 exhibited stable recombination suppressing properties down to 1% in F2 and F3 (χ^2^ = 904.14; df = 1; p < < 0.001), with a sample size of more than 4000 and 5000 mosquitos screened, respectively.

**Table 2:** Recombination frequencies between *w* and the M locus in 32 iso-male lines

Line	F	Genotypes					Recombination frequency
		Parental		Recombinant		Total	
		wt males	we females	we males	wt females		
**5**	**F2**	**607**	**599**	**6**	**11**	**1223**	**0.0139**
	**F3**	**834**	**392**	**11**	**10**	**1253**	**0.014**
6	F2	699	498	122	92	1411	0.151
9	F2	323	306	48	61	738	0.147
10	F2	741	652	91	92	1576	0.116
16	F2	633	69	138	571	1411	0.147
17	F2	578	611	70	63	1322	0.101
19	F2	664	284	35	27	664	0.093
31	F2	620	676	157	131	1584	0.181
34	F2	405	462	43	44	954	0.091
**35**	**F2**	**2267**	**2022**	**30**	**13**	**4332**	**0.010**
	**F3**	**2548**	**2597**	**33**	**19**	**5197**	**0.010**
37	F2	340	253	33	24	650	0.088
41	F2	342	335	49	51	777	0.128
43	F2	209	173	34	41	457	0.164
45	F2	478	402	45	31	956	0.079
48	F2	191	198	33	36	458	0.151
50	F2	306	255	35	32	628	0.107
51	F2	559	364	47	21	991	0.068
52	F2	347	474	38	67	926	0.113
54	F2	549	497	56	50	1152	0.092
55	F2	438	459	53	40	990	0.094
58	F2	243	242	17	18	520	0.067
59	F2	185	165	25	26	401	0.128
60	F2	416	228	46	18	708	0.090
61	F2	40	20	9	7	76	0.211
64	F2	443	178	34	19	674	0.079
67	F2	302	270	14	21	607	0.058
	F3	575	492	50	71	1188	0.102
68	F2	435	320	54	29	838	0.099
71	F2	393	202	30	23	648	0.082
78	F2	348	112	46	13	519	0.114
82	F2	314	191	32	16	553	0.087
91	F2	334	400	44	75	853	0.140
93	F2	379	289	51	25	744	0.102

In bold: lines showing promising recombination-suppressing properties between *w* and *M* loci

### Searching of naturally-occurring recombination suppressors (re - M and w - M)

To identify naturally-occurring recombination suppressors among *re* and the M locus, males from sixteen wild type strains were crossed with homozygous *re* mutant virgin females ‘*en masse*’. All F1 males exhibited the wild type phenotype and were backcrossed to virgin *re* females. Recombination frequencies were measured for one (F2) or two (F2 and F3) generations. Despite the presence of varying levels of recombination, there was no combination leading to an important recombination suppression (Additional file 2 Table S2). Following the same approach, we searched for naturally-occurring inversions that could act as recombination suppressors by crossing homozygous *w* mutant females with males from fourteen different genomic backgrounds. Again, although varying recombination frequencies were observed between the two loci (*w - M*) no combination could severely reduce recombination (Additional file 3 Table S3).

## Discussion

### Main findings

Induction of inversions was attempted through irradiation at 30 Gy. Analysis of recombination frequencies revealed at least one line that suppressed recombination between *re* and the M locus (line 35) and at least two lines that suppressed recombination between *w* and the M locus (lines 5 and 35). Searching for naturally-occurring mutations in many different genomic backgrounds did not result in any combination that efficiently suppressed recombination either between *re* and *M* or *w* and M.

*Recombination in the re-M-w region*: Different studies up to now have assessed recombination between these markers and all agree that the M locus resides between *re* and *w* (Additional file 4 Table S4)*.* Recombination in the *re-M* region has been shown to vary between 1 and 12 cM, but usually in the more narrow range of 2.5–8.5 cM [45, 47, 61–66]. Data from our group generated from recombination frequencies from consecutive generations using the same strains (BRA and Red-eye) suggest that recombination ranges between 1 and 2.5% [36]. At the same time, most studies suggest that recombination in the *w-M* region varies between 13 and 19 cM [44, 64–68] while our baseline recombination data collected from multiple generations using the same strains (BRA and Higgs White-eye) indicate that recombination ranges between 9 and 13% [36]. Variation in recombination frequencies have been attributed to factors such as the age, the sex, and the temperature, with the presence of widespread (small or extended) chromosomal rearrangements being the most probable explanation [45, 47, 61, 62, 68]. Following irradiation, most of the iso-male families screened exhibited recombination frequencies within the expected range. Line 35 significantly suppressed recombination both in the *re -*M and the *w*-M regions, whereas line 5 significantly suppressed recombination only in the *w-*M region, pointing to the presence of different inversions. Such low recombination frequencies (less than 0.2% for the *re-*M and less than 2% for the *w-*M region) have not been described before pointing to these lines as irradiation-induced inversions. Additional cytogenetic and/or genomic work can shed light to the genomic organization of chromosome I in these lines. It is encouraging that these two lines have been maintained without problems since they were isolated, suggesting that the chromosomal rearrangements involved do not have a severe negative effect on fitness.

## Conclusions

SIT, IIT, and combined approaches, are receiving renewed interest for vector control (mosquitoes). Male recombination is restricting the development of GSS through classical genetics, since the desirable mutations must be very closely linked to the M locus. Inducing inversions through irradiation is a feasible strategy to isolate recombination suppressors either on the M or the m chromosome for *Aedes* species. Such inversions can be incorporated in genetic sexing strains to enhance their genetic stability and support vector control strategies that aim to population suppression through male-delivered sterility.

## Methods

### Strains description and rearing conditions

The Red Eye, Higgs White Eye (HWE), and a Brazilian wild-type strain (BRA) were used in the present study. The “wild-type” color of the eye in *Ae. aegypti* is dark brown/black and stable during all developmental stages. The color of the eye in the Red Eye strain (*re*) is constantly red during all developmental stages, although it darkens with age. The color of the eye in the Higgs White Eye strain (*w*) is white and stable in all developmental stages, although it darkens with age. *Ae. aegypti* strains were maintained in the insectary of the Insect Pest Control Laboratory (Joint FAO/IAEA Division, Seibersdorf, Austria) at 27 ± 1 °C, 80% relative humidity and a photoperiod of 12/12 h day/night. Adult mosquitoes were kept in standard (30 × 30 × 30 cm) insect rearing plastic cages (BugDorm-41,515 insect cage) with constant access to a 10% sucrose solution. Blood feeding of adult female mosquitoes was performed using porcine blood three times per week. Moistened oviposition papers (white Creped Filter Papers) were inserted into the cages 48 h after the last blood feeding round in order to collect the mosquito eggs.

### Irradiation

Wild type male pupae of the ‘BRA’ strain were irradiated in batches of 100 at 30 Gy, 30 to 36 h post pupation, using the method described as a stackable petri dish canister, in a Gammacell 220, self-shielded, gamma-ray Cobalt 60 irradiator [69]. Dosimetry was performed according to standard operating procedures regarding dosimetry systems for SIT [70] and all readings were within the 95% confidence intervals. The irradiated male pupae emerged in BugDorm-1 cages (30x30x30 cm), with access to 10% sucrose solution.

### Crosses

Crosses are presented in Fig. 1 and followed the experimental set up described in the past [60] for the isolation of recombination suppressors on the M chromosome.

#### Isolation of recombination suppressors (re - M)

Parental cross was performed ‘*en masse*’. Virgin homozygous *re* mutant females were transferred in the cages with the irradiated ‘BRA’ males, in a ratio of 3:1 (approximately 150 females for 50 males). F1 males were separated and screened in respect to the eye color at the pupal stage. All F1 progeny exhibited the wild type eye phenotype. One hundred F1 males were individually placed in BugDorm cages (BD4S1515: 15 cm × 15 cm × 15 cm) and backcrossed to five homozygous *re* mutant virgin females. F2 progeny were sorted based on sex and eye color at the pupal stage. Parental (males with wild type eyes and females with red eyes) and recombinant (males with red eye and females with black eye) genotypes were recorded. All families were kept through inbreeding after the removal of the recombinant genotypes, at the pupal stage (Additional file 5 Fig. S1). Promising families (showing evidence of reduced recombination) were upscaled and recombination frequencies were recorded in subsequent generations as described for F2 generation. χ^2^ statistics were used to compare the observed recombination frequencies of the F2 and F3 of the promising families against the recombination frequency observed in the BRA genetic background (Additional file 2 Table S2). Calculations were performed using the Microsoft Excel 2016 formulas.

#### Isolation of recombination suppressors between (w - M)

Since resulting families represented iso-male lines, five F2 males from each family were transferred to new cages and crossed with ten homozygous *w* mutant virgin females. This is considered as the parental cross for this experiment. We acknowledge that genetic recombination may influence the genomic background of these males preventing them from being ‘genetically identical’. However, the fact that these two mutations cannot be simultaneously tested due to the inconclusive phenotypes of double mutants led us to the compromise of sequentially testing them. F1 progeny exhibited the expected, wild type phenotype, and 5–10 F1 males were backcrossed with approximately 25 homozygous *w* mutant virgin females. F2 progeny were sorted based on sex and eye color at the pupal stage. Sex was also verified at the adult stage (Additional file 5 Fig. S1). Parental (males with wild type eyes and females with white eyes) and recombinant (males with white eye and females with black eye) genotypes were recorded. All families were kept through inbreeding after the removal of the recombinant genotypes at the pupal stage. Promising families (showing evidence of reduced recombination) were upscaled and recombination frequencies were recorded in F3. χ^2^ statistics were used to compare the observed recombination frequencies of the F2 and F3 of the promising families against the recombination frequency observed in the BRA genetic background (Additional file 3 Table S3). Calculations were performed using the Microsoft Excel 2016 formulas.

#### Isolation of naturally-occurring recombination suppressors (re - M and w - M)

Since naturally-occurring mutations have been reported in different mosquito species, males from sixteen wild type colonized populations were crossed with virgin females of the ‘Red Eye’ and the ‘Higgs White Eye’ strains, respectively. Parental crosses were performed ‘*en masse*’ with 5–10 males and approximately 20–25 females. F1 progeny exhibited the expected, wild type phenotype, and at least ten F1 males were backcrossed with 25 virgin females of the respective homozygous mutant strain (either *re* or *w*). F2 progeny were sorted based on sex and eye color at the pupal stage. Parental (males with wild type eyes and females with either white or red eyes) and recombinant (males with either white or red eye and females with black eye) genotypes were recorded. All strains were kept through inbreeding after the removal of the recombinant genotypes at the pupal stage. Promising strains (showing evidence of reduced recombination) were upscaled and recombination frequencies were recorded in the following generation (F3).

## Supplementary Information

**Additional file 1: Table S1**: Egg production of 100 iso-male lines.

**Additional file 2: Table S2**: Recombination frequencies between *re* and the M locus in 16 different genomic backgrounds.

**Additional file 3: Table S3**: Recombination frequencies between *we* and the M locus in 14 different genomic backgrounds.

**Additional file 4: Table S4**: Recombination frequencies between *re, we,* and the M/m locus from previous studies.

**Additional file 5: Figure S1**: Identification of parental and recombinant genotypes in the F2 generation.

## Data Availability

All data generated during this study are included in this published article and its supplementary information files.

## Abbreviations

SIT: Sterile insect technique
IIT: Incompatible insect technique
*re*: the red eye gene
*w*: the white eye gene
M: the male determining locus

## References

[1] Guzman MG, Harris E (2015). Dengue. Lancet.

[2] Shepard DS, Undurraga EA, Betancourt-Cravioto M, Guzmán MG, Halstead SB, Harris E (2014). Approaches to refining estimates of global burden and economics of dengue. PLoS Negl Trop Dis.

[3] Diagne CT, Weaver SC, Dia I, Knight R, Guerbois M, Faye O (2014). Vector competence of Aedes aegypti and Aedes vittatus (Diptera: Culicidae) from Senegal and Cape Verde archipelago for west African lineages of Chikungunya virus. Am J Trop Med Hygiene.

[4] Li MI, Wong PSJ, Ng LC, Tan CH (2012). Oral susceptibility of Singapore Aedes (Stegomyia) aegypti (Linnaeus) to Zika virus. PLoS Negl Trop Dis.

[5] Garcia R, Marchette NJ, Rudnick A (1969). Isolation of Zika virus from Aedes Aegypti mosquitoes in Malaysia *. Am J Trop Med Hygiene..

[6] Auguste AJ, Lemey P, Pybus OG, Suchard MA, Salas RA, Adesiyun AA (2010). Yellow fever virus maintenance in Trinidad and its dispersal throughout the Americas. J Virol.

[7] Beasley DWC, McAuley AJ, Bente DA (2015). Yellow fever virus: genetic and phenotypic diversity and implications for detection, prevention and therapy. Antivir Res.

[8] Simmons CP, Farrar JJ, van Vinh CN, Wills B (2012). Dengue. N Engl J Med.

[9] Mousson L, Dauga C, Garrigues T, Schaffner F, Vazeille M, Failloux A-B (2005). Phylogeography of Aedes (Stegomyia) aegypti (L.) and Aedes (Stegomyia) albopictus (Skuse) (Diptera: Culicidae) based on mitochondrial DNA variations. Genet Res.

[10] Halstead SB (2012). Dengue vaccine development: A 75 solution?. Lancet.

[11] Sabchareon A, Wallace D, Sirivichayakul C, Limkittikul K, Chanthavanich P, Suvannadabba S (2012). Protective efficacy of the recombinant, live-attenuated, CYD tetravalent dengue vaccine in Thai schoolchildren: a randomised, controlled phase 2b trial. Lancet.

[12] Lima EP, Paiva MHS, de Araújo AP, da Silva ÉVG, da Silva UM, de Oliveira LN (2011). Insecticide resistance in Aedes aegypti populations from Ceará, Brazil. Parasites Vectors.

[13] Vontas J, Kioulos E, Pavlidi N, Morou E, della Torre A, Ranson H (2012). Insecticide resistance in the major dengue vectors Aedes albopictus and Aedes aegypti. Pestic Biochem Physiol.

[14] Moyes CL, Vontas J, Martins AJ, Ng LC, Koou SY, Dusfour I (2017). Contemporary status of insecticide resistance in the major Aedes vectors of arboviruses infecting humans. PLoS Negl Trop Dis.

[15] Egger JR, Ooi EE, Kelly DW, Woolhouse ME, Davies CR, Coleman PG (2008). Reconstructing historical changes in the force of infection of dengue fever in Singapore: implications for surveillance and control. Bull World Health Organ.

[16] Ooi E-E, Goh K-T, Gubler DJ (2006). Dengue prevention and 35 years of vector control in Singapore. Emerg Infect Dis J.

[17] Ranson H, Burhani J, Lumjuan N, Black WC (2010). Insecticide resistance in dengue vectors. TropIKA.net.

[18] O’Connor L, Plichart C, Sang AC, Brelsfoard CL, Bossin HC, Dobson SL (2012). Open release of male mosquitoes infected with a Wolbachia biopesticide: field performance and infection containment. PLoS Negl Trop Dis.

[19] Bourtzis K, Dobson SL, Xi Z, Rasgon JL, Calvitti M, Moreira LA (2014). Harnessing mosquito-Wolbachia symbiosis for vector and disease control. Acta Trop.

[20] Bourtzis K, Lees RS, Hendrichs J, Vreysen MJB (2016). More than one rabbit out of the hat: radiation, transgenic and symbiont-based approaches for sustainable management of mosquito and tsetse fly populations. Acta Trop.

[21] Gilles JRL, Schetelig MF, Scolari F, Marec F, Capurro ML, Franz G (2014). Towards mosquito sterile insect technique programmes: exploring genetic, molecular, mechanical and behavioural methods of sex separation in mosquitoes. Acta Trop.

[22] Kyrou K, Hammond AM, Galizi R, Kranjc N, Burt A, Beaghton AK (2018). A CRISPR–Cas9 gene drive targeting doublesex causes complete population suppression in caged Anopheles gambiae mosquitoes. Nat Biotechnol.

[23] Kandul NP, Liu J, CHMS, Wu SL, Marshall JM, Akbari OS (2019). Transforming insect population control with precision guided sterile males with demonstration in flies. Nat Commun.

[24] Zheng X, Zhang D, Li Y, Yang C, Wu Y, Liang X (2019). Incompatible and sterile insect techniques combined eliminate mosquitoes. Nature..

[25] Papathanos PA, Bossin HC, Benedict MQ, Catteruccia F, Malcolm CA, Alphey L (2009). Sex separation strategies: past experience and new approaches. Malar J.

[26] Papathanos PA, Bourtzis K, Tripet F, Bossin H, Virginio JF, Capurro ML, et al. A perspective on the need and current status of efficient sex separation methods for mosquito genetic control. Parasites Vectors. 2018;11. 10.1186/s13071-018-3222-9. 10.1186/s13071-018-3222-9 PMC6304774 30583720

[27] Zacarés M, Salvador-Herranz G, Almenar D, Tur C, Argilés R, Bourtzis K, et al. Exploring the potential of computer vision analysis of pupae size dimorphism for adaptive sex sorting systems of various vector mosquito species. Parasit Vectors. 2018;656(11 Suppl 2):560–72. 10.1186/s13071-018-3221-x PMC6304766 30583722

[28] Franz G, Dyck VA, Hendrichs J, Robinson AS (2005). Genetic sexing strains in Mediterranean fruit Fly, an example for other species amenable to large-scale rearing for the sterile insect technique BT - sterile insect technique: principles and practice in area-wide integrated Pest management.

[29] Kittayapong P, Ninphanomchai S, Limohpasmanee W, Chansang C, Chansang U, Mongkalangoon P (2019). Combined sterile insect technique and incompatible insect technique: the first proof-of-concept to suppress Aedes aegypti vector populations in semi-rural settings in Thailand. PLoS Negl Trop Dis.

[30] Kittayapong P, Kaeothaisong N-O, Ninphanomchai S, Limohpasmanee W (2018). Combined sterile insect technique and incompatible insect technique: sex separation and quality of sterile *Aedes aegypti* male mosquitoes released in a pilot population suppression trial in Thailand. Parasit Vectors.

[31] Bond JG, Osorio AR, Avila N, Gómez-Simuta Y, Marina CF, Fernández-Salas I (2019). Optimization of irradiation dose to Aedes aegypti and Ae albopictus in a sterile insect technique program. PLOS ONE.

[32] Bouyer J, Culbert NJ, Dicko AH, Pacheco MG, Virginio J, Pedrosa MC, et al. Field performance of sterile male mosquitoes released from an uncrewed aerial vehicle. Science Robotics. 2020;5. 10.1126/scirobotics.aba6251. 10.1126/scirobotics.aba6251 33022616

[33] WHO, IAEA. TDR | guidance framework for testing the sterile insect technique as a vector control tool against Aedes-borne diseases. WHO. 2020. http://www.who.int/tdr/publications/year/2020/guidance-framework-for-testing-SIT/en/. Accessed 27 Apr 2020.

[34] Bouyer J, Yamada H, Pereira R, Bourtzis K, Vreysen MJB. Phased conditional approach for mosquito management using sterile insect technique. Trends Parasitol. 2020;36:325–36. 10.1016/j.pt.2020.01.004 32035818

[35] Dyck VA, Hendrichs JP, Robinson AS (2005). Sterile insect technique: principles and practice in area-wide integrated Pest management.

[36] Koskinioti P, Augustinos AA, Carvalho DO, Misbah-ul-Haq M, Pillwax G, Duran de la Fuente L, et al. Genetic sexing strains for the population suppression of the mosquito vector *Aedes aegypti*. Philos Transact R Soc B Biol Sci. 2020;in press. 10.1098/rstb.2019.0808. 10.1098/rstb.2019.0808 PMC7776939 33357054

[37] Carvalho DO, Torres-Monzon JA, Koskinioti P, Wijegunawardana NDAD, Liang X, Pillwax G, et al. *Aedes aegypti* lines for combined sterile insect technique and incompatible insect technique applications: the importance of host genomic background. Entomologia Experimentalis et Applicata. 2020;n/a n/a. 10.1111/eea.12892.

[38] McClelland GAH. Sex-linkage in Aedes aegypti. Trans roy Soc trop Med Hyg. 1962;56:4.

[39] Newton ME, Southern DI, Wood RJ (1974). X and Y chromosomes of *Aedes aegypti* (L.) distinguished by Giemsa C-banding. Chromosoma.

[40] Hall AB, Basu S, Jiang X, Qi Y, Timoshevskiy VA, Biedler JK (2015). A male-determining factor in the mosquito *Aedes aegypti*. Science.

[41] Timoshevskiy VA, Severson DW, Debruyn BS, Black WC, Sharakhov IV, Sharakhova MV. An integrated linkage, chromosome, and genome map for the yellow fever mosquito *Aedes aegypti*. PLoS Neglected Trop Dis. 2013;7:article e2052. 10.1371/journal.pntd.0002052. 10.1371/journal.pntd.0002052 PMC3573077 23459230

[42] Aryan A, Anderson MAE, Biedler JK, Qi Y, Overcash JM, Naumenko AN, et al. Nix alone is sufficient to convert female Aedes aegypti into fertile males and myo-sex is needed for male flight. PNAS. 2020. 10.1073/pnas.2001132117. 10.1073/pnas.2001132117 PMC7395513 32661163

[43] Hall AB, Timoshevskiy VA, Sharakhova MV, Jiang X, Basu S, Anderson MAE (2014). Insights into the preservation of the homomorphic sex-determining chromosome of Aedes aegypti from the discovery of a male-biased gene tightly linked to the M-locus. Genome Biol Evol.

[44] Bhalla SC (1968). White eye, a new sex-linked mutant of Aedes aegypti. Mosquito News.

[45] Bhalla SC, Craig GB (1970). Linkage analysis of chromosome 1 of Aedes aegypti. Can J Genet Cytol.

[46] Ouda NA, Wood RJ. Inheritance of brown-eye and colourless-eye in the mosquito *Aedes aegypti*. Ann Trop Med Parasitol. 1983;77:211-18. 10.1080/00034983.1983.11811699. 10.1080/00034983.1983.11811699 6882068

[47] Ouda NA, Wood RJ. Variation in recombination M/m-re in three strains of mosquito _*Aedes aegypti*_ (L.). J Biol Sci Res. 1985;16:153–73.

[48] Brown AW (1967). Mechanisms and inheritance of resistance and selection of resistance potential in Aedes aegypti. Bull World Health Organ.

[49] Klassen W, Brown AWA (1964). Genetics of insecticide-resistance and several visible mutants in Aedes Aegypti. Can J Genet Cytol.

[50] Bhalla SC, Craig GB (1967). Bronze, a female-sterile mutant of Aedes aegypti. J Med Entomol.

[51] Craig GB, Gillham NW (1959). The inheritance of larval pigmentation in Aedes aegypti. J Hered..

[52] Bernhardt SA, Blair C, Sylla M, Bosio C, Black WC (2009). Evidence of multiple chromosomal inversions in Aedes aegypti formosus from Senegal. Insect Mol Biol.

[53] Timoshevskiy VA, Kinney NA, de Bruyn BS, Mao C, Tu Z, Severson DW (2014). Genomic composition and evolution of *Aedes aegypti* chromosomes revealed by the analysis of physically mapped supercontigs. BMC Biology.

[54] Stevison LS, Hoehn KB, Noor MAF (2011). Effects of inversions on within- and between-species recombination and divergence. Genome Biol Evol..

[55] Noor MAF, Grams KL, Bertucci LA, Reiland J (2001). Chromosomal inversions and the reproductive isolation of species. PNAS..

[56] Kirkpatrick M, Barton N (2006). Chromosome inversions, Local Adaptation and Speciation. Genetics.

[57] Fisher K, Caceres C. A filter rearing system for mass reared genetic sexing strains of Mediterranean fruit fly (Diptera: Tephritidae). In: Area-wide Control of Fruit Flies and other Insect Pests. Eds. Keng-Hong Tan. Penerbit University Sains Malaisia; 2000. p. 543–50.

[58] Augustinos AA, Targovska A, Cancio-Martinez E, Schorn E, Franz G, Cáceres C (2017). Ceratitis capitata genetic sexing strains: laboratory evaluation of strains from mass-rearing facilities worldwide. Entomologia Experimentalis et Applicata..

[59] Zacharopoulou A, Augustinos AA, Drosopoulou E, Tsoumani KT, Gariou-Papalexiou A, Franz G (2017). A review of more than 30 years of cytogenetic studies of Tephritidae in support of sterile insect technique and global trade. Entomologia Experimentalis et Applicata.

[60] Bhalla SC (1973). Sex-linked crossover suppressors in the mosquito, *Aedes aegypti*. Mosquito News.

[61] MacDonald WW, Sheppard PM (1965). Cross-over values in the sex chromosomes of the mosquito Aedes aegypti and evidence for the presence of inversions. Ann Trop Med Parasitol.

[62] Hickey WA, Craig GBJ (1966). Genetic distortion of sex ratio in a mosquito, *Aedes aegypti*. Genetics.

[63] McClelland GAH (1966). Sex-lnkage at two loci affecting eye pigment in the mosquito Aedes aegypti (Diptera: Culicidae). Can J Genet Cytol.

[64] Petersen JL, Larsen JR, Craig GBJ (1976). Palp-antenna, a homeotic mutant in Aedes aegypti. J Hered.

[65] Munstermann LE, Craig GB (1979). Genetics of Aedes aegypti: updating the linkage map. J Hered..

[66] Pearson AM (1980). Linkage studies on the mutant short-wing in the mosquito Aedes aegypti. Heredity..

[67] Bhalla SC (1970). Paracentric inversions and detection of sex linked recessive lethals in aedes aegypti. Can J Genet Cytol.

[68] Dickson LB, Sharakhova MV, Timoshevskiy VA, Fleming KL, Caspary A, Sylla M (2016). Senegalese Aedes aegypti ( L ) is associated with chromosome rearrangements. PLoS Negl Trop Dis.

[69] Helinski MEH, Parker AG, Knols BGJ. Radiation-induced sterility for pupal and adult stages of the malaria mosquito Anopheles arabiensis. Malar J. 2006;5. 10.1186/1475-2875-5-41. 10.1186/1475-2875-5-41 PMC1475870 16700906

[70] IAEA. Dosimetry System for SIT: Manual for Gafchromic® film. IAEA; 2004. http://www-naweb.iaea.org/nafa/ipc/public/Dosimetry_SOP_v11.pdf.

