# Supplementary material for: Irradiation induced inversions suppress recombination between the M locus and morphological markers in Aedes aegypti
Source: BMC Genet. 2020 Dec 18;21(Suppl 2):142. doi: 10.1186/s12863-020-00949-w (PMC7747368; doi:10.1186/s12863-020-00949-w)
Supplement: Supplementary file 1 — Additional file 1: Table S1: Egg production of 100 iso-male lines. [file 12863_2020_949_MOESM1_ESM.docx]

**Additional File 1 Table S1: Egg production of 100 iso-male lines**

| Line | GC1 | GC2 | GC3 | Total | Line | GC1 | GC2 | GC3 | Total | Line | GC1 | GC2 | GC3 | Total |
| --- | --- | --- | --- | --- | --- | --- | --- | --- | --- | --- | --- | --- | --- | --- |
| **1** | 0 | 0 | 0 | 0 | **35** | 189 | 153 | 62 | 404 | **68** | 69 | 0 | 0 | 69 |
| **2** | 0 | 0 | 0 | 0 | **36** | 0 | 0 | 0 | 0 | **69** | 0 | 0 | 0 | 0 |
| **3** | 0 | 0 | 0 | 0 | **37** | 47 | 60 | 0 | 107 | **70** | 0 | 0 | 0 | 0 |
| **4** | 10 | 4 | 0 | 14 | **38** | 0 | 0 | 0 | 0 | **71** | 0 | 42 | 0 | 42 |
| **5** | 87 | 1 | 0 | 88 | **39** | 0 | 0 | 0 | 0 | **72** | 45 | 0 | 0 | 45 |
| **6** | 63 | 0 | 0 | 63 | **40** | 43 | 0 | 0 | 43 | **73** | 0 | 0 | 0 | 0 |
| **7** | 25 | 0 | 0 | 25 | **41** | 136 | 0 | 0 | 136 | **74** | 0 | 0 | 0 | 0 |
| **8** | 5 | 0 | 0 | 5 | **42** | 71 | 0 | 0 | 71 | **75** | 0 | 14 | 0 | 14 |
| **9** | 0 | 75 | 0 | 75 | **43** | 65 | 72 | 54 | 191 | **76** | 0 | 0 | 0 | 0 |
| **10** | 63 | 0 | 0 | 63 | **44** | 0 | 0 | 0 | 0 | **77** | 0 | 0 | 0 | 0 |
| **11** | 12 | 0 | 0 | 12 | **45** | 85 | 0 | 0 | 85 | **78** | 84 | 86 | 0 | 170 |
| **12** | 0 | 0 | 0 | 0 | **46** | 0 | 0 | 0 | 0 | **79** | 0 | 0 | 0 | 0 |
| **13** | 0 | 14 | 0 | 14 | **47** | 0 | 0 | 0 | 0 | **80** | 0 | 0 | 0 | 0 |
| **14** | 0 | 0 | 0 | 0 | **48** | 226 | 0 | 0 | 226 | **81** | 0 | 0 | 0 | 0 |
| **15** | 0 | 0 | 0 | 0 | **49** | 108 | 0 | 0 | 108 | **82** | 0 | 32 | 0 | 32 |
| **16** | 85 | 0 | 0 | 85 | **50** | 51 | 18 | 0 | 69 | **83** | 35 | 0 | 0 | 35 |
| **17** | 54 | 0 | 0 | 54 | **51** | 117 | 0 | 0 | 117 | **84** | 0 | 0 | 0 | 0 |
| **18** | 0 | 0 | 0 | 0 | **52** | 115 | 15 | 64 | 194 | **85** | 0 | 0 | 0 | 0 |
| **19** | 82 | 16 | 0 | 98 | **53** | 0 | 7 | 0 | 7 | **86** | 0 | 0 | 0 | 0 |
| **20** | 41 | 0 | 0 | 41 | **54** | 167 | 89 | 0 | 256 | **87** | 0 | 0 | 0 | 0 |
| **21** | 16 | 0 | 0 | 16 | **55** | 129 | 0 | 0 | 129 | **88** | 0 | 0 | 0 | 0 |
| **22** | 0 | 0 | 0 | 0 | **56** | 0 | 0 | 0 | 0 | **89** | 0 | 0 | 0 | 0 |
| **23** | 0 | 0 | 0 | 0 | **57** | 65 | 67 | 0 | 132 | **90** | 0 | 0 | 0 | 0 |
| **24** | 57 | 36 | 0 | 93 | **58** | 85 | 0 | 0 | 85 | **91** | 0 | 52 | 0 | 52 |
| **25** | 59 | 53 | 0 | 112 | **59** | 106 | 44 | 0 | 150 | **92** | 0 | 0 | 0 | 0 |
| **26** | 0 | 0 | 0 | 0 | **60** | 121 | 43 | 47 | 211 | **93** | 65 | 0 | 0 | 65 |
| **27** | 15 | 41 | 0 | 56 | **61** | 129 | 84 | 47 | 260 | **94** | 35 | 0 | 0 | 35 |
| **28** | 4 | 0 | 0 | 4 | **62** | 0 | 0 | 0 | 0 | **95** | 0 | 0 | 0 | 0 |
| **29** | 0 | 0 | 0 | 0 | **63** | 21 | 12 | 0 | 33 | **96** | 0 | 0 | 0 | 0 |
| **30** | 0 | 0 | 0 | 0 | **64** | 0 | 76 | 69 | 145 | **97** | 0 | 0 | 0 | 0 |
| **31** | 45 | 0 | 0 | 45 | **65** | 0 | 18 | 0 | 18 | **98** | 0 | 35 | 0 | 35 |
| **32** | 46 | 0 | 0 | 46 | **66** | 0 | 0 | 0 | 0 | **99** | 0 | 0 | 0 | 0 |
| **33** | 75 | 0 | 0 | 75 | **67** | 48 | 0 | 0 | 48 | **100** | 0 | 0 | 0 | 0 |
| **34** | 132 | 145 | 76 | 353 |  |  |  |  |  |  |  |  |  |  |
|  |  |  |  |  | **average1**  **(*st. dev*)** | 34.33  (*49.3*) | 14.04  (*30.1*) | 4.19  (*15.6*) | 52.56  (*78.2*) | **average2**  **(*st. dev*)** | 60.23  (*52.0*) | 24.63  (*36.6*) | 7.35  (*20.2*) | 92.21  (*84.1*) |
