# Supplementary material for: Irradiation induced inversions suppress recombination between the M locus and morphological markers in Aedes aegypti
Source: BMC Genet. 2020 Dec 18;21(Suppl 2):142. doi: 10.1186/s12863-020-00949-w (PMC7747368; doi:10.1186/s12863-020-00949-w)
Supplement: Supplementary file 2 — Additional file 2: Table S2: Recombination frequencies between re and the M locus in 16 different genomic backgrounds. [file 12863_2020_949_MOESM2_ESM.docx]

**Table S2: Recombination frequencies between *re* and the M locus in 16 different genomic backgrounds**

| **Strain status** | **Genomic background** | **F** | **Genotypes** | | | | | **Recombination frequency** |
| --- | --- | --- | --- | --- | --- | --- | --- | --- |
|  |  |  | **Parental** | | **Recombinant** | | **Total** |  |
|  |  |  | **wt males** | **re females** | **re males** | **wt females** |  |  |
| Recently colonized populations | Argentina_1 | F2 | 281 | 238 | 2 | 7 | 528 | 0.017 |
|  | Argentina_2 | F2 | 240 | 198 | 7 | 15 | 460 | 0.048 |
|  | Bahamas | F2 | 163 | 67 | 4 | 3 | 237 | 0.029 |
|  | Brazil | F2 | 242 | 230 | 3 | 6 | 481 | 0.018 |
|  | Costa Rica | F2 | 306 | 335 | 16 | 18 |  | 0.050 |
|  | Cuba | F2 | 234 | 27 | 10 | 0 | 271 | 0.037 |
|  | Indonesia | F2 | 19 | 13 | 0 | 5 | 37 | 0.278 |
|  |  | F3 | 306 | 119 | 8 | 184 | 617 | 0.311 |
|  | Jamaica | F2 | 333 | 284 | 9 | 5 | 631 | 0.022 |
|  | Mexico | F2 | 422 | 221 | 9 | 13 | 665 | 0.033 |
|  | Singapore | F2 | 1076 | 804 | 33 | 17 | 1930 | 0.026 |
|  |  | F3 | 435 | 269 | 9 | 3 | 734 | 0.016 |
|  | Sri Lanka | F2 | 401 | 377 | 7 | 5 | 790 | 0.015 |
|  | Thailand | F2 | 62 | 61 | 4 | 5 | 132 | 0.068 |
|  |  | F3 | 734 | 698 | 42 | 44 | 1518 | 0.057 |
| old laboratory strains | IB12 | F2 | 540 | 313 | 36 | 22 | 911 | 0.064 |
|  |  | F3 | 468 | 443 | 22 | 16 | 949 | 0.040 |
|  | Liverpool | F2 | 690 | 726 | 14 | 14 | 1444 | 0.019 |
|  |  | F3 | 266 | 178 | 16 | 8 | 468 | 0.051 |
|  | Rockefeller | F2 | 562 | 397 | 47 | 31 | 1037 | 0.075 |
|  |  | F3 | 389 | 378 | 18 | 19 | 804 | 0.046 |
|  | Waco | F2 | 466 | 401 | 86 | 46 | 999 | 0.132 |
|  |  | F3 | 290 | 274 | 29 | 22 | 615 | 0.083 |
