# Supplementary material for: Irradiation induced inversions suppress recombination between the M locus and morphological markers in Aedes aegypti
Source: BMC Genet. 2020 Dec 18;21(Suppl 2):142. doi: 10.1186/s12863-020-00949-w (PMC7747368; doi:10.1186/s12863-020-00949-w)
Supplement: Supplementary file 3 — Additional file 3: Table S3: Recombination frequencies between we and the M locus in 14 different genomic backgrounds. [file 12863_2020_949_MOESM3_ESM.docx]

**Additional File 3Table S3: Recombination frequencies between *we* and the M locus in 14 different genomic backgrounds**

| **Genomic background** | **F** | **Genotypes** | | | | | **Recombination frequency** |
| --- | --- | --- | --- | --- | --- | --- | --- |
|  |  | **Parental** | | **Recombinant** | | **Total** |  |
|  |  | **wt males** | **re females** | **re males** | **wt females** |  |  |
| Argentina_1 | F2 | 307 | 206 | 62 | 42 | 617 | 0.168 |
| Argentina_2 | F2 | 136 | 180 | 20 | 26 | 362 | 0.127 |
| Bahamas | F2 | 417 | 344 | 97 | 54 | 912 | 0.166 |
| Brazil | F2 | 329 | 317 | 35 | 40 | 721 | 0.104 |
| Costa Rica | F2 | 200 | 214 | 38 | 30 | 482 | 0.141 |
| Cuba | F2 | 227 | 189 | 44 | 44 | 504 | 0.174 |
| Jamaica | F2 | 187 | 133 | 22 | 5 | 347 | 0.078 |
| Mexico | F2 | 63 | 35 | 11 | 5 | 114 | 0.140 |
| Singapore | F2 | 669 | 595 | 112 | 98 | 1474 | 0.142 |
| Sri Lanka | F2 | 89 | 87 | 12 | 11 | 199 | 0.116 |
| IB12 | F2 | 482 | 422 | 87 | 66 | 1057 | 0.145 |
|  | F3 | 274 | 286 | 30 | 48 | 638 | 0.122 |
| Liverpool | F2 | 240 | 150 | 17 | 94 | 501 | 0.221 |
|  | F3 | 311 | 144 | 34 | 137 | 626 | 0.273 |
| Rockefeller | F2 | 207 | 150 | 25 | 16 | 398 | 0.103 |
|  | F3 | 283 | 81 | 100 | 414 | 878 | 0.206 |
| Waco | F2 | 336 | 280 | 67 | 37 | 720 | 0.144 |
|  | F3 | 330 | 335 | 47 | 65 | 777 | 0.144 |
