# Supplementary material for: Irradiation induced inversions suppress recombination between the M locus and morphological markers in Aedes aegypti
Source: BMC Genet. 2020 Dec 18;21(Suppl 2):142. doi: 10.1186/s12863-020-00949-w (PMC7747368; doi:10.1186/s12863-020-00949-w)
Supplement: Supplementary file 4 — Additional file 4: Table S4: Recombination frequencies between re, we, and the M/m locus from previous studies. [file 12863_2020_949_MOESM4_ESM.docx]

**Additional File 4 Table S4: Recombination frequencies between *re, we,* and the M/m locus from previous studies**

| Study | Year | Genetic linkage (cM) | | Notes on recombination | Reference |
| --- | --- | --- | --- | --- | --- |
| MacDonald and Seppard | 1965 | *re - m* | 2.1-5.7 | Evidence for the presence of inversions | [61] |
| Hickey and Craig | 1966 |  | 6.7-7.8 | Strain specific differences. Crosses involving males from sex ratio distortion lines exhibited reduced recombination (1-3 cM) | [62] |
| McClelland | 1966 |  | 6.4 |  | [63] |
| Bhalla and Craig | 1970 |  | 7.2-8.4 | Heterogeneity of results: inversions, male age, temperature, sex-specific | [45] |
| Petersen et al. | 1976 |  | 4 |  | [64] |
| Munstermann and Craig | 1979 |  | 7-12 | In the ‘standard’ genetic linkage map | [65] |
| Pearson | 1980 |  | 2.5 |  | [66] |
| Ouda et al | 1985 |  | ~6-8 | Variation between strains, and random effect of generations. D may suppress recombination. Possibility of inversions | [47] |
| Koskinioti et al | 2020 |  | 1-2.5 | fluctuation across generations | [36] |
| Bhalla | 1968 | *w - m* | 17 |  | [44] |
| Bhalla | 1970 |  | 15 |  | [67] |
| Petersen et al. | 1976 |  | 13-18 |  | [64] |
| Munstermann and Craig | 1979 |  | 14-19 | In the ‘standard’ genetic linkage map | [65] |
| Pearson | 1980 |  | 14.7 |  | [66] |
| Dickson et al | 2016 |  | ranging between zero and unlinked | evidence for widespread chromosomal rearrangements | [68] |
| Koskinioti et al | 2020 |  | 9-13 | fluctuation across generations | [36] |
