# Supplementary material for: Irradiation induced inversions suppress recombination between the M locus and morphological markers in Aedes aegypti
Source: BMC Genet. 2020 Dec 18;21(Suppl 2):142. doi: 10.1186/s12863-020-00949-w (PMC7747368; doi:10.1186/s12863-020-00949-w)
Supplement: Supplementary file 5 — Additional file 5: Figure S1: Identification of parental and recombinant genotypes in the F2 generation. [file 12863_2020_949_MOESM5_ESM.docx]

**Additional File 5 Figure S1: Identification of parental and recombinant genotypes in the F2 generation**

**
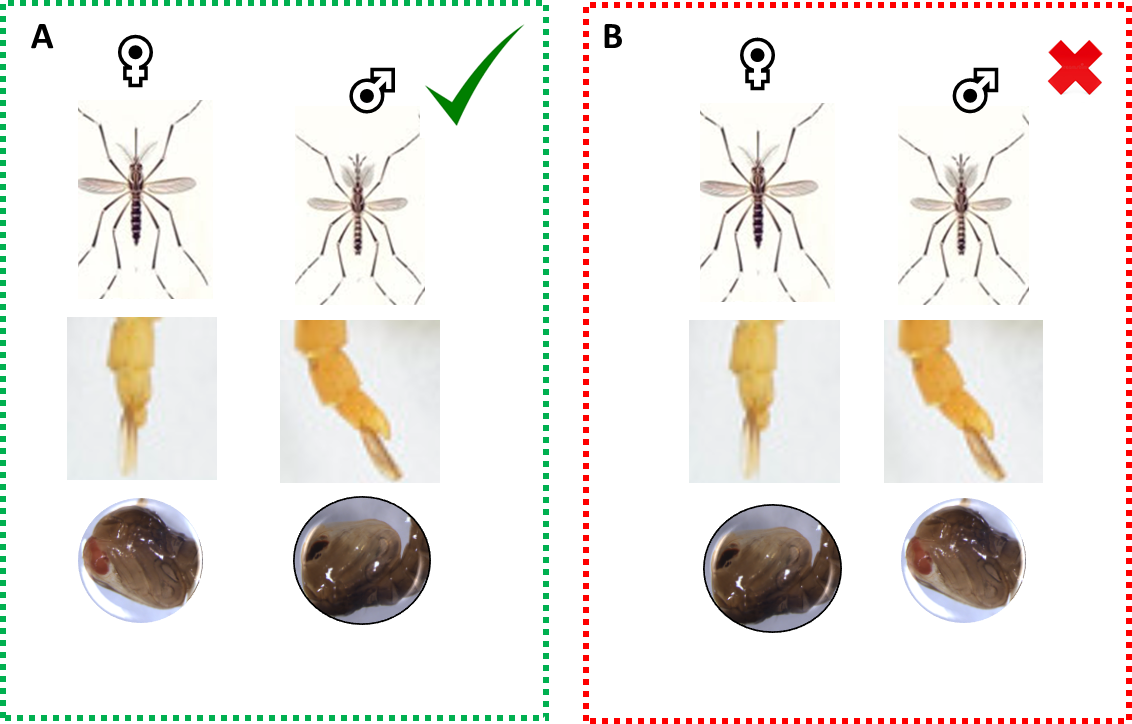
**

A: The green panel indicates the parental genotypes (homozygous *re* mutant females and wild type males). B: The red panel indicates the recombinant genotypes (wild type females and homozygous *re* mutant males). Eye color and sex were screened at early pupal stage. Sex was verified also at adult stage. The same approach was followed with the *w* mutation. The sex-specific characters used were the structure of the genital lobe at the pupa stage and the plumose antennae at the adult stage.
